# Measurement of the Developing Foot in Shod and Barefoot Paediatric Populations: A Narrative Review

**DOI:** 10.3390/children9050750

**Published:** 2022-05-19

**Authors:** Maisie Squibb, Kelly Sheerin, Peter Francis

**Affiliations:** 1 Department of Science and Health, South East Technological University, Kilkenny Road, Moanacurragh, R93 V960 Carlow, Ireland; peter.francis@itcarlow.ie; 2 Sports Performance Research Institute New Zealand (SPRINZ), School of Sport and Recreation, Auckland University of Technology, 1010 Auckland, New Zealand; kelly.sheerin@aut.ac.nz

**Keywords:** development, footwear, paediatrics, medial longitudinal arch, hallux valgus angle, foot shape, muscle strength, musculoskeletal

## Abstract

The theory that footwear may change foot shape dates back 100 years. Since this period, research has revealed the anatomical and functional consequences that footwear can cause to the foot. Children’s feet remain malleable as they undergo developmental changes until adolescence, which is why childhood is arguably a crucial period to understand how footwear can affect natural foot development. This review explored the development of the foot in children and adolescents and the methods used to measure the different foot structures; it comments on the key issues with some of these methods and gives direction for future research. Various internal and external factors can affect foot development; the main factors are age, gender, ethnicity, body mass index (BMI) and footwear habits. Research on how footwear can affect foot development has increased over the years and the final section of this review aimed to unpick the findings. Studies investigating the influence of footwear habits on foot length and width have established inconsistent findings. Many of the studies in the review did not control for internal and external factors that can affect foot development. There was also a limited number of studies that investigated hallux valgus angle and muscle strength differences in those with different footwear habits. Moreover, multiple studies in the final section of this review did not successfully examine the footwear habits of the participants and instead used observations or self-assessments, which is a major limitation. Future research should examine footwear behaviors and other confounding factors when investigating the development of the foot in children and adolescents. Moreover, researchers should critically evaluate the methods used to quantify the different structures of the foot to ensure valid and reliable parameters are being used.

## 1. Introduction

Humans have been wearing footwear for approximately 40,000 years [1]. Footwear was initially minimal in design and developed to protect the foot from harsh environments. Modern footwear represents a transition from using footwear for utility toward functional and fashion-related purposes. In the past century, footwear has changed drastically in shape; cushioned and supportive soles, narrow toe boxes and increased heel elevation are just some characteristics common in modern-day conventional footwear [2].

The influence of footwear on foot morphology was first noted over 100 years ago [1]. Flatter feet, greater hallux valgus angles, and decreased foot widths are just some of the characteristics associated with conventional footwear use in adults. However, unlike adult feet, children’s feet remain malleable as they undergo developmental changes until adolescence, which is why childhood is a crucial period to understand how footwear can affect natural foot development. Children can build strong and resilient feet during these early years, or foot problems and deformities can arise.

Previous meta-analyses and systematic and narrative reviews have focused on the effects of footwear on various foot, body and gait characteristics [3,4,5,6,7]. Shod gait has been associated with an increase in stride length, a greater dorsiflexion angle at foot ground contact, as well as a faster walking speed [3,4,5,6]. Authors have established an increase in tibialis anterior muscle activity, and a decrease in the intrinsic motion of the foot while participants walk in shoes [3,4,6]. Wegener et al. [4] reported reductions in eight of the nine variables measuring foot motion in shoes compared to barefoot conditions, with only ‘subtalar’ rotation increasing in the shod children. Authors have reported increased foot width when under load in children walking barefoot compared to walking in shoes or sandals [3,4,5]. A more even distribution of pressure is experienced when walking barefoot together with a reduced initial impact force compared with those wearing shoes [3,5]. There is contrasting evidence concerning the morphological characteristics of the foot—more specifically, regarding the medial longitudinal arch [3,5,7]. Some of the literature has indicated a higher incidence of flat foot amongst shod children, whilst one study reported that footwear had little influence on the height of the medial longitudinal arch [3,5,7]. Minimalist style footwear, defined as footwear free of any support or cushioning, produced negligible differences in gait kinematics to walking barefoot [3,6]. Some research suggests that flexible footwear may inhibit the foot less than conventional shoes [5]. It remains a matter of debate whether minimalist style footwear is a better match for our feet; it may lower the risk of injury and deformity compared to more supportive conventional shoes. 

The last review conducted by Morrison et al. [5] in 2018 briefly looked at the literature (<2017) concerning the developmental effects of footwear but chose to focus on the medial longitudinal arch. The foot is a complex structure that must function as a rigid lever during locomotion and be pliable for shock absorption. To achieve this functional adaptability, the bones, muscles and tendons in the foot must work in synergy and therefore the measurement of other structures in the foot should also be considered. There is no definitive method for quantifying the structures within the foot and therefore considerations need to be made when synthesizing and comparing the literature. Altered foot function can lead to impaired gait, muscle imbalances, balance problems and a range of pathologies affecting the lower limbs. Therefore, understanding the anatomy of the foot is important for understanding its functional role. Many intrinsic and extrinsic factors influence the development of the foot, including age, gender, body weight, activity levels and footwear habits. The main body of literature investigating footwear habits involves adults with completely developed feet. It is necessary to investigate whether the same trends are seen in the developing feet of children. Since 2017, key studies have investigated footwear habits and their effects on foot development in children and adolescents, and this necessitates an up-to-date review to appraise and synthesize more recent findings. The outcomes of this narrative review will be valuable for future research to identify any shortcomings of the literature concerning footwear’s effects on foot development in children and adolescents. 

The overall aim of this review is to describe the development of the foot through childhood and investigate the intrinsic and extrinsic factors that may affect this. A secondary aim is to discuss the measurement techniques used and comment on their construct validity. This review is split into three main sections: The first section (Section 2) describes the development of the structures in the foot throughout childhood and into adolescence. The second section (Section 3) discusses different measurements of the developing foot, commenting on the validity of the measurement techniques used and the intrinsic and extrinsic factors that may influence this. The final section (Section 4) looks at footwear as an extrinsic factor that may influence foot development in children and adolescents. Finally, directions for future research will be given based on the literature reviewed.

## 2. Structural Development

### Bone and Soft Tissue Maturation

The maturation of the bony structures in the foot refers to both form (size and shape) and the hardening of the bone [8]. At birth, children’s feet consist primarily of soft tissue, and complete ossification of the bony structures occurs throughout the first ten years of life [8,9,10,11]. This process begins in the foot between the third and fifth prenatal months [8,12]. Ossification of the hindfoot also commences prenatally, starting at the calcaneus and followed by the talus, talar and cuboid [13]. There is variance in the research regarding what age navicular ossification occurs, ranging from girls aged between 18 months and two years and boys aged between two and three and a half years old [14,15]. Ossification of the growth plates begins within the first ten years, and closure of these plates happens at the end of growth, between the ages of 15 and 21 years [13]. 

Soft tissue structures such as muscles, tendons and connective tissues are noticeable in newborns; yet, complete stiffness of these structures is not reached until adulthood [8,12,16]. Strengthening of the muscles occurs after a child is born, with the application of gravity and motion [12,17]. Consolidation of the soft tissues in the foot begins between two and four years, and complete maturation is not reached until adolescence [13]. 

The development of feet encompasses the maturation of both the bony and soft tissue structures. The results of this maturation are observed through changes in foot measures such as foot length and width. This information highlights how children’s feet remain malleable until adolescence; therefore, this is a crucial period for understanding how external factors such as footwear may affect the developing foot. The studies which have investigated the maturation of these structures contain large sample sizes and use valid and reliable methods which, overall, contribute to the strength of their findings.

## 3. Measurements of the Developing Foot

### 3.1. Foot Length

Changes in the dimensions of a child’s foot have been widely reported in the literature [18,19,20,21,22,23,24,25,26,27,28,29,30,31,32,33]. Foot length is the most noticeable change as the foot develops, especially in young children [18,19,20,21,22,23,24,25,26,27,28,29,30,31,32,33]. The methods for collecting foot length data varies within the literature. Some authors measure from the most posterior aspect of the calcaneus to the top of the great toe [20,31,32,33], while others measure to the top of the most protruding toe [24,29]. Although there is a variation in the data collection methods, the findings are typically concurrent and, therefore, either can be used.

Both cross-sectional and longitudinal types of studies have described the development of foot length in children and adolescents (Table 1). At birth, the foot is around one-third of its final length [34], and within the first three to five years of life rapid growth occurs, and approximately two-thirds of the final foot length is achieved [24,25,29,33]. Hereafter, an average annual increase of between 0.8 and 1 cm is typically observed from age 5 to 12 in girls and 5 to 14 in boys [22,23]. These increases are concurrent with other literature that has reported no significant differences in foot length between boys and girls before 12 years old [20,28,32]. After the age of 12, the differences in average length between girls and boys increases precipitously; girls feet change very little after this age (an average of 0.7 cm), whereas boys have an average of 2.6 cm additional length by the point of maturity [20,24,29]. At the age of 14, 75% of girls’ feet finish growing and reach a mature length [24]. Boys’ feet continue to grow until 16 years of age, where 70% reach a mature length [24]. Similar findings have reported that girls’ foot lengths grow very little after 13 years old, and boys’ feet grow very little after 15 years old [20].

### 3.2. Foot Width

Changes in foot width (also referred to as foot breadth, ball of foot width or forefoot width) have been well described in the literature [18,21,23,24,25,26,27,28,29,30,31,32,33] (Table 1). There are multiple methods for collecting foot width data. Some measure the broadest aspect of the foot [18], whilst other researchers measure the width at defined anatomical landmarks (first metatarsophalangeal joint to the fifth metatarsophalangeal joint) [31]. A range of digital tools [31], Harris mats [19], sketches [27], calipers [18] and custom-made footboards [24,31] have been used to measure foot width.

All studies mentioned in this paragraph used the maximum forefoot width method to describe foot width. Foot width increased with age (Table 1) from an average of 5.7 cm in infants to 8.9 cm in 13-year-olds [28]. These findings were similar to Cheng et al. [32], who concluded that girls had a mean width of 5.9 cm at age three, which increased to 8.3 cm at age 13. At age nine, girls had a wider foot width than those of the opposite gender (8.1 cm compared to 7.9 cm); however, between the ages of 10–12, boys presented with wider feet (8.5–9 cm compared to 8.2–8.9 cm) [18]. The growth rate in foot width in girls plateaued at age 14; however, a residual amount of growth was seen in boys until the age of 18 (0.2–0.3 cm) [26]. At the end of growth, a mean difference of approximately 1 cm was reported between girls and boys foot width, with boys having wider feet than girls [26].

### 3.3. Medial Longitudinal Arch

The medial longitudinal arch is an intricate structure comprised of muscles, bones, tendons and ligaments which lock and unlock, giving the foot its ability to be both pliable for shock absorption and a rigid lever for the push-off phase of walking [35,36,37]. An increase or decrease in the height of the arch—referred to as the pes cavus and pes planus, respectively—can impair the function of the medial longitudinal arch, leading to gait abnormalities, misalignment and muscular imbalance [38,39,40,41]. Infants are born with flat feet, and throughout childhood, the medial longitudinal arch develops [29,37,42,43]. Initially, a fat pad underneath the medial longitudinal arch covers the plantar surface of the foot, making the arch invisible to the eye [44,45,46,47]. As infants progress to standing and walking, resolution of the fat pad begins and continues until approximately five years old [21,43,48]. 

Many methods have been used to evaluate the medial longitudinal arch structure (Table 2). The most common methods include the arch index [21,28,46,49,50,51,52,53], Clarke’s/footprint angle [37,49,54,55,56,57,58,59], the Chippaux-Smirak index [37,49,54,57,60], Staheli’s index [57,60,61] and the arch height ratio [20,62]. Parameters that are calculated through footprints and anthropometric equipment offer a non-invasive and inexpensive alternative to the digital systems used in clinical practice [63]. However, footprint and anthropometric methods do have some limitations, such as the inaccuracy of their measurements and complications in interpretation [64].

Although the studies mentioned in the following section did not use the same measurements, they all depicted a similar pattern of foot development across the early age span. Table 3 presents the key studies and findings related to the longitudinal arch development through childhood and adolescence. The following section describes the development of the arch measured with these different assessments.

#### 3.3.1. Arch Index

Arch index was quantified using footprint data. This method uses the ratio of the area of the middle third of the footprint to the entire footprint area when standing (not including the toes) [65]. Cavanagh et al. [65] classified the arch heights into high, normal and low arches, whereby an arch index of 0.21 or less indicated a high arch, greater than 0.21 to less than 0.26 a normal arch and 0.26 or greater a low arch (flatter foot). Studies that have used arch index to characterize the medial longitudinal arch in children are consistent in their findings [19,21,28,66]. The highest arch index values are present in the youngest children, decreasing with age as the foot develops [19,21,28,66]. Research shows that children under one year have an arch index of between 0.32 and 0.40 [19,28,66]. Muller et al. [28] reported that the fastest rate of change is seen between the ages of one and six (0.32 to 0.20). This aligns with Bosch et al. [66] who reported that arch index values decrease from 0.40 in one-year-olds to 0.27 in six-year-olds. Muller et al. [28] and Bosch et al. [66] found that at age seven, arch index values range from 0.18 to 0.19—similar to index values seen in adults [19]. From this age, arch index values plateau [19,28,66].

#### 3.3.2. Clarke’s/Footprint Angle

There was a lack of consensus in terms of the name of this measurement, but for this review, ‘Clarke’s/footprint angle’ was used. Clarke’s/footprint angle uses footprint data to quantify the height of the medial longitudinal arch [37,67]. A straight line is projected onto a footprint from the most medial aspect of the border from the heel and forefoot (A) [37]. A second line is projected from the medial forefoot border to the apex of the concavity of the arch of the footprint (B) [37]. The angle between line A and B represents the Clarke’s/Footprint Angle (C). For this review, research based on Jaworski et al. [68] classifications of the Clarke’s/footprint angle were included. A Clarke’s/footprint angle of 42° was the lowest limit of a normal arch height. Values of 0–29.9° were considered flat; 30–34.9° showed a fallen/low arch and 35–41.9° were termed intermediary [56]. 

Although Clarke’s/footprint angle values differed in the literature, there was an agreement regarding arch development [37,58,59]. Three studies agreed that Clarke’s/footprint angle increases with age [37,58,59]. Forriol et al. [37] reported that between 68 and 72% of girls’ and 74% of boys’ feet are flat in the age group between three and four years. This was in agreement with Jankowicz-Szymanska et al. [58,59], who reported that the mean angle of all participants aged between three and six years was below the ‘normal arch’ values, indicating that they had flat feet. A single study investigated the Clarke’s/footprint angle in children over seven years old; a greater frequency of Clarke’s/footprint angle values of 42° and above were apparent in the older age groups (age 9–11: 55–56%; 12–14: 42–69%; 15–17: 52–66%) [37]. 

#### 3.3.3. Chippaux–Smirak Index

Quantified using a footprint, the Chippaux–Smirak index is created from a straight line drawn along the medial border of the footprint between the heel and forefoot’s most medial points [37]. A second line (B) is drawn from the medial forefoot border for the maximal width of the forefoot print [37]. A final line (C) is drawn parallel to this, which represents the minimum width of the foot around the arch [37]. Line C is divided by line B and multiplied by 100 to give a percentage. The Chippaux–Smirak index incorporates five categories to describe the arch. A score of 0% indicates a high arch foot; an index of 0.1–29.9% represents a standard arched foot; 30–39.9% represents an intermediary arch; 40–44.9% represents a lowered arch and a flat foot is indicated by an index of 45% or more [68]. 

Two studies quantified the medial longitudinal arch using the Chippaux–Smirak index. Forriol et al. [37] found that children between three- and four-years old present with the highest frequency of flat feet, with values of between 45–52%; the Chippaux–Smirak index values then decrease gradually with age up to 11 years in boys and 12 years in girls [37]. Between the ages of 9 and 11, the frequency of those with a normal arch rises to 59–77% [37]. Nikolaidou et al. [56] reported that only 13% of those aged between 9 and 11 years old have a normal arch and 79% have an arch below normal. 

#### 3.3.4. The Staheli Index

As with the Chippaux–Smirak index, the Staheli index is quantified using a footprint [69]. The width of the foot at the arch (A) is divided by the width of the heel (B) [69]. Staheli et al. [69] and Ozlem et al. [61] both used a score of between 0.3 and 1.0 to describe a normal range in adults and children after middle childhood and a range of 0.7–1.35 in infants. However, Onodera et al. [36] considered values between 0.44 and 0.89 as normal.

The Staheli index decreases with age up until age 12, suggesting the development of and increase in arch height [61]. In children aged between three and 10, the Staheli index decreases from a mean of 0.8 in three-year-olds to 0.5 in ten-year-olds [36]. Ozlem et al. [61] found a similar pattern, with the greatest proportion of feet with a low arch in the youngest age category. Onodera et al. [36] reported that most children aged between 3- and 10-years old present with an arch within the normal range (73% of three-year-olds). There is, however, a lack of research using the Staheli index to quantify the expected arch development in children.

#### 3.3.5. Arch Height Ratio

The arch height ratio, presented as a percentage, is used to assess the integrity of the medial arch without the need for expensive equipment. The arch height ratio (navicular height/foot length × 100) has been used to quantify the arch height of children in only two studies [20,62]. The researchers agreed that values increased with age, outlining the development and growth of the arch; however, no normative values have been reported [20,62]. Waseda et al. [20] described a stable arch height ratio in children aged between 6 and 10 years (13.8–13.6%), with little change noted. A more sizeable increase was apparent in girls and boys aged between 11 and 18 years (14.3–16.9%). Morita et al. [62] described an arch height ratio of 14.8% in boys and 14.4% in girls aged eight and nine years old. In children aged between 10 and 11, an arch height ratio of 13.7% in boys and 14.7% in girls was seen [62]. Waseda et al. [20] found that the ratio plateaued at age 17 in girls and 18 in boys. 

### 3.4. Measurement of the Developing Foot Summary and Remarks

Foot length and foot width developed similarly with age. Comparable growth trends were evident, with both measurements increasing with age in boys and girls. At age nine, girls had wider feet than boys. Between the ages of 10 and 12, boys’ foot width surpassed girls’. Growth rates plateaued in girls around the age of 14, but residual growth in boys remained until the age of 18. Foot length growth was similar in boys and girls until the age of 12, when girls’ growth plateaued. Boys’ feet continued to grow longer until around 16 years old. 

The sample sizes used in the research concerning the development of foot length and width were mostly large, which contributes to the reliability of the findings. The use of longitudinal study designs is effective in uncovering growth patterns over time, which is a major advantage compared to cross-sectional study designs. A major limitation of the studies examining foot length and width development was the lack of intrinsic and extrinsic factors that may influence the data. Ethnic growth patterns have been detected after controlling for nutrition, environment, maternal and childcare, income distribution, gross domestic product, health services and political factors [70,71,72,73]. For example, Asiatic mean height values for 7-year-olds are lower than European, African and Latin-American populations [73]. As a result of this, to truly understand growth patterns, research should include and measure participants’ ethnicity to explain variations in foot anthropometrical data. The references in Table 1 did not include data on participant ethnicity, and as such, one can only speculate. Many of these references denote the country in which the participants are from; however, there could be numerous ethnicities in a country. Sex, age and weight were commonly measured to uncover how these factors effect foot length and width.

There was agreement across all reviewed studies that arch height increased throughout childhood; however, it remains unclear when development ends. Most low-arched feet were found in the youngest age categories and these numbers tended to decrease through childhood. Dissimilar to this, Onodera et al. [36] reported that 73% of three-year-olds already had a normal arch. Arch index assessments found the most significant rate of change within children aged between one and six years old [19,21,28,66]. Contrasting these findings, studies that used arch height ratio to quantify arch development reported notable changes between 10 and 13 years old. The plateau in development differed drastically between the different methods and ranged from 6 years up to 18 years.

Numerous assessments were used to characterize the medial longitudinal arch; however, none are recognized as the ‘gold standard’ method. The authors believe that many of these assessments have been created to quantify the structure with equipment available to the researchers. Practically, this may mean that researchers and practitioners without access to advanced equipment can quantify the medial longitudinal arch. However, there is a lack of clarity regarding assessment classification systems and a lack of large-scale research studies, leading to discrepancies and gaps within specific age groups. In addition, due to the large number of methods for characterizing the arch, comparisons between different assessments are complicated and often not possible. Consequently, a valid and reproducible method for quantifying the arch of children is needed and a consensus on its assessment must be established.

**Table 3:** A summary of the studies examined relating to the development of the medial longitudinal arch.

Author	Study Type	Participants & Age Range	Mean Age (±SD)	Method of Analysis	Main Findings
Volpon et al. [29]	Cross-sectional	338 male 334 female 0–15 years	N/A	Footprint contact index II	During the first two years of life, the values for arch height were the largest (indicating a flatter arch). Values decreased rapidly until the age of 6 years, where they slowed until the age of 10.
Bosch et al. [19]	Longitudinal	16 male 20 girls 0–10 years	14.6 ± 1.8 months 122.8 ± 2 months	Arch index	Arch index values decreased by 44% between the ages of 0 and 10 years, indicating the development of the arch. Substantial development of the arch from a flat foot to a regular foot was seen through the ages. By the age of 10, inter-individual differences were equivalent to adults.
Onodera et al. [36]	Cross-sectional	391 male and female 3–10 years	N/A	Arch index Chippaux–Smirak Index Staheli index	The frequency of low arched feet was higher at the age of three compared to other ages. An increase in longitudinal arch development was seen (as measured by all indices). After six years old, arch maturation continued slowly until the age of 10.
Müller et al. [28]	Cross-sectional	3738 male 4050 female 1–13 years	7.2 ± 2.9 years	Arch index	The arch index declined during growth. The mean arch index ranged from 0.3 in infants to 0.2 in 13-year-olds. Between the ages of one and six, a considerable reduction in the arch index was seen; however, from age six, it plateaued. Arch index values were comparable to those of adult’s feet by the age of 6–7.
Mickle et al. [21]	Cross-sectional	36 boys 52 girls 3–5 years	4.2 ± 0.6 years	Arch index	Boys had a higher arch index value than girls (lower arch). Arch index values ranged between 0.01 and 0.36. The flatter feet of boys were thought to be due to a thicker midfoot fat pad. Resolution and development of the arch started earlier in girls.
Pfeiffer et al. [9]	Cross-sectional	424 male 411 female 3–6 years	N/A	Visual inspection Position of the heel	44% of children had a flexible flat foot. The prevalence of flat feet decreased with age; in 3-year-old children, 54% presented with flat feet; 24% of 6-year-olds had flat feet. The prevalence of flat foot was higher in boys (52%) than girls (36%). The prevalence of flat foot continued to decrease from 71% to 32% from 3–6 years.
Forriol et al. [37]	Cross-sectional	663 male 1013 female 3–17 years	N/A	Chippaux–Smirak Index Footprint angle	A high percentage of flat feet was present between 3 and 4 years with the footprint angle (B: 73.5%; G: 68.4–71.9%). Footprint angle increased with age (arch height increased with age) (B 3–4 years: 21.7°; B 15–17 years: 44.6°; G 3–4 years: 26.1°; G 15–17 years: 46.8°). Chippaux–Smirak Index decreased with age (arch height increased with age; B 3–4 years: 50.5%; B 15–17 years: 30.1%; G 3–4 years: 46.9%; G 15–17 years: 27.4%).
Jankowicz-Szymanska et al. [58]	Cross-sectional	710 males 654 female 3–7 years	N/A	Clarke’s angle	Clarke’s angle increased (increase in arch height) with age, apart from in 6-year-old girls. Girls of all ages had higher arches than boys. Clarke’s angle was highest in those with a healthy body weight.
Jankowicz-Szymanska et al. [59]	Longitudinal	102 males 105 females 3–6 years	N/A	Clarke’s angle	A smaller Clarke’s angle (lower arch height) was seen in boys compared to girls. At age six, boys had a lower longitudinal arch than girls (B: 36.63 ± 13.09°; G: 37.31 ± 11.74°).
Ozlem et al. [61]	Cross-sectional	299 male 280 females 6–12 years	9.23 ± 1.66 years	Staheli arch index	The mean arch index for all children was 0.74 ± 0.25. The mean arch index of group 1 (normal to mild flatfoot) was 0.67 ± 0.19. The mean arch index of group 2 (moderate to severe flatfoot) was 1.12 ± 0.17. A negative correlation between the arch index and age (increase in arch height with age).
Waseda et al. [20]	Cross-sectional	5311 male 4844 female 6–18 years	N/A	Arch height ratio	There was no gender difference in arch height ratio. In boys, the arch height ratio was relatively flat until 11 years old but increased quickly between age 11–13 (B 11 years: 14.1 ± 2.6%; B 13 years: 15.4 ± 2.6%). In girls, the arch height ratio was flat until 10 years old but significantly rose between 10–12 years old (G 10 years: 13.6 ± 2.5%; G 12 years: 14.8 ± 2.6%). A plateau in girls was seen around the age of 17 (16.5 ± 2.3%) and in boys at age 18 (16.9 ± 2.4%).
Stavlas et al. [74]	Cross-sectional	2935 male 2931 6–17 years	N/A	Footprint angle Arch index Chippaux–Smirak Index	Boys had higher rates of low arched feet compared to girls (B 6 years: 9% low/flat arch; G 6 years: 7.2% low/flat arch). The frequency of high arched and low arched feet present in the youngest age group decreased with age (B 6 years 9% low/flat arch; B 17 years: 0.6% low/flat arch; G 6 years: 7.2% low/flat arch; G 17 years: 1.6% low/flat arch).
Morita et al. [62]	Cross-sectional	146 male 155 female 8–11 years	8.6 ± 0.5 years 10.6 ± 0.5 years	Arch height ratio	Arch height was greater in older girls than in boys of the same age (B: 13.7 ± 2.3; G: 14.7 ± 2.4).
Nikolaidou et al. [56]	Cross-sectional	67 male 65 female 9–11 years	10.4 ± 0.9 years	Chippaux–Smirak Index Arch index	Arch index measurements showed that 30% of participants presented with a low foot type. Chippaux–Smirak Index measurements showed that 46% of participants presented with a low or flat foot type.
Staheli et al. [69]	Cross-sectional	441 male and female 1–80 years	N/A	Staheli index	During infancy, typical values ranged between 0.7 and 1.35. A sizeable normal range from about 0.3–1 is seen after the middle of childhood and through to adulthood.
Bosch et al. [66]	Cross-sectional	104 male and female 1–70 years	1.3 ± 0.4 years 7 ± 0.4 years	Arch index	The largest arch index values were found in the toddler group (0.36), which described a flat arch. The 7-year-old group had an arch index of 0.18. Adults presented with a mean arch index of 0.19 and seniors 0.21.

Abbreviations: B—Boys; G—Girls; N/A—Not Available.

## 4. The Influence of Footwear on Foot Development

The previous section discussed the development of foot length, width and medial longitudinal arch height during childhood and adolescence. It also revealed that these structures can be affected by various internal and external factors—namely, gender, age, BMI, physical activity levels, ethnicity and footwear habits. The main limitation evident within the previous section was the lack of controls for these confounding factors. We understand that controlling these intrinsic and extrinsic factors can be time consuming; however, we feel it is necessary to truly understand the growth and development of the foot in children. One factor that was absent from the studies was footwear habits. Due to this, we can only speculate that the data was collected from shoe-wearing populations. 

The following section focuses on footwear habits and foot development in children and adolescents. A child’s foot is sensitive to the actions exerted upon it, and therefore, it is essential that children’s footwear protects the foot and facilitates its development. Shoe styles for children tend to mimic adult fashion trends, especially concerning shape. However, children’s feet are distinctly different to those of adults. For example, the epiphyseal union of all long bones occurs during late adolescence, as does the increase in mechanical stiffness and stability of the ligaments and tendons. This next section aims to uncover the differences in foot structure between shod and barefoot child populations. 

### 4.1. Foot Width

Few studies have investigated the differences in foot width in children with different footwear habits and those who wear poorly fitted footwear [50,75,76]. Hollander et al. [50] and Kusumoto [76] investigated populations with different footwear habits, whilst Wolf et al. [75] investigated acute changes when changing footwear conditions. Overall, both Hollander et al. [50] and Kusumoto [76] found that foot width was similar in populations with different footwear habits. However, Hollander et al. [50] did report that barefoot participants aged between 6 and 10 have wider feet than their shod counterparts (Table 4). Hollander et al. [50] compared the foot morphology of 810 barefoot and shod children aged 6 to 18 years old. The study classified a participant barefoot with a three-point Likert scale which scored a child based on how often they were barefoot in a series of locations and settings. Participants were classified as barefoot if they scored ≥3 from a maximum of six points [50]. Kusumoto [76] compared rural Filipino children with urban children from Tokyo aged between 7 and 18 years old [76]. The Filipino children did not wear restrictive footwear, 97% wore rubber sandals, and 3% were barefoot, whereas children from Tokyo wore either athletic shoes (87%) or leather shoes (15%) [76]. Wolf et al. [75] found that foot width was acutely reduced in children when wearing shoes. The foot width variability whilst barefoot was almost 10%, whereas, in the two shoe conditions, it was only 6% and 4%, respectively [75]. The author of this paper hypothesized that this was due to a width limitation caused by the shoes [75]. Ethnicity, BMI, physical activity levels and gender were all factors controlled for by Hollander et al. [50]. Conversely, it is unclear which factors Kusumoto [76] included in their study. Gender, age and footwear use were clearly detailed; however, the author mentioned the poor protein intake in the agricultural regions of the Philippines where some of the participants were from but did not investigate whether this influenced the data.

### 4.2. Foot Length

Foot length in children has been studied among different countries, including South Africa, Germany, Japan, the Philippines and Australia [49,50,76]. Kusumoto [76] and Mauch et al. [49] both found that barefoot children had shorter feet than their shod counterparts (Table 4). Kusumoto et al. [76] found significant differences in boys aged between 8 and 12 and girls aged between 7 and 11. Mauch et al. [49] established significant differences in preschool-aged children aged between three and five. On the other hand, Hollander et al. [50] found that barefoot children had longer feet than their shoe-wearing counterparts between the ages of 6 and 10 and 14–18. It should be noted that Mauch et al. [49] did not investigate the footwear habits of the participants but assumed that the Australian participants would walk more frequently barefoot or in open footwear. Hollander et al. [50] did not find any significant effects of confounders on foot length and both Kusumoto [76] and Mauch et al. [49] did not investigate the effects of possible confounding factors. 

Previous research in adults uncovered that when relativized to body length, habitually barefoot groups show larger feet than habitually shod groups [7,77,78]. None of these studies controlled for confounding variables. Taking the conflicting evidence regarding foot length into consideration, currently we cannot say for certain what effects footwear use has on foot length. Future work should collect data on footwear use and duration as well as factors such as gender, BMI, ethnicity and physical activity levels.

### 4.3. Foot Musculature

Weak intrinsic foot muscles have been implicated in various foot disorders such as flat feet, hallux valgus, claw toe and hammertoe in children [79,80]. In adults, evidence suggests that being barefoot or wearing minimal shoes free of arch support improves intrinsic and extrinsic foot muscle strength [81,82,83,84]. To date, no research studies have tested the hypothesis that spending time barefoot or in minimal shoes can strengthen the intrinsic foot muscles in children; however, many have speculated about the effect that footwear has on foot strength [50,80,83,85,86,87]. 

A single study has investigated foot strength in populations with different footwear habits [86]. Aibast et al. [86] found that habitually barefoot adolescents have stronger feet during short foot exercises than their habitually shod counterparts. No significant differences were noted in hallux flexor muscle strength between barefoot and shod adolescents; however, when the first four flexor digit muscles were compared, habitually barefoot participants were stronger than their shod counterparts [86]. Aibast et al. [86] did acknowledge that a limitation of their study was the fact that they relied on self-reported identification of whether the participant was barefoot or shod. When controlled, age, gender and BMI did not affect foot strength in this study [86]. The effect of physical activity levels on foot strength remained unclear [86].

### 4.4. Medial Longitudinal Arch 

Arch height was the most prevalent foot characteristic researched across populations with different footwear habits [42,49,50,57,85,86,88]. Research was consistent in showing shod participants as having flatter feet than those who are barefoot (Table 5). Mauch et al. [49] compared arch height in children from two different continents. The study found that German preschool children aged between three and five years displayed flatter feet than their Australian counterparts, who were thought to be barefoot more often [49]. Similarly, a longitudinal study conducted by Tong et al. [85] found that children between seven and nine years old who wore closed-toe shoes displayed a lower arch than those who wore sandals or slippers. A study of children and adolescents aged between 6 and 18 years old reported an increase in static arch height in those who predominantly grew up barefoot [50]. These results are consistent with Rao et al. [42], who reported a low prevalence of flat foot among unshod participants aged between 4 and 13 years. The higher incidence of flat foot (lower arch height) within the shod individuals suggests that shoe-wearing predisposes a person to flat feet [42]. When looking at children aged six and under, 17% of those who are shod present with flat foot, yet only 9% of unshod children do so [42]. In both the shod and unshod groups, the prevalence of flat foot progressively decreases with age [42,57]. Barefoot adolescents aged between 12 and 18 have higher arches compared to their shod counterparts [86]. A retrospective study conducted by Sachithanandam et al. [88] found a higher prevalence of flat foot in adults who began using footwear in early childhood [88]. The prevalence of flat foot was 3.24% in subjects who started wearing shoes before the age of five years, 3% in those aged between 6 and 15 years and 2% in those who started wearing shoes aged 16 years or over [88]. This study suggests there is a connection between wearing shoes in early childhood and flat foot.

Hollander et al. [50] and Sachithanandam et al. [88] found that BMI had a significant effect on arch height in children, whilst Rao et al. [42] did not. Mauch et al. [49] took a different approach and matched the two populations for BMI. Mauch et al. [49] did not investigate the footwear habits of the participants and only speculated about them. Tong et al. [85], Sachithanandam et al. [88] and Hollander et al. [50] used a survey to uncover the footwear habits of the participants. Rao et al. [42] observed and commented on the habits of participants. While observations are a cost-effective way of determining the footwear habits of the participants on the day, without asking them or their parents, we do not know their behaviors outside of the testing setting. The authors of this review feel this is a major limitation of studies investigating footwear habits. Ethnic differences were controlled for only by Hollander et al. [50], who found no significant differences. Mauch et al. [49] and Tong et al. [85] did not mention ethnicity, which is a limitation of these studies. 

### 4.5. Hallux Valgus Angle

Characterized by the progressive valgus angulation of the first metatarsophalangeal joint, hallux valgus is a common foot deformity [89,90,91,92]. Hallux valgus has been linked to foot pain [92,93,94] and impaired gait parameters [75,89] in children and adolescents. Radiographic imaging is often used to directly measure hallux valgus deformities, indicating the relative position between the hallux and the first metatarsal [95]. The normative values of the hallux angle are between 10 and 15°, and anything over this is deemed a mild, moderate or severe deformity [96]. For ethical and methodological reasons, radiographic imaging is not always feasible, so alternative, less invasive methods are used [97,98].

Research suggests that habitually barefoot individuals have a reduced hallux angle compared to shod individuals [93,94]. Wearing ill-fitting footwear in terms of width and length has been associated with an increased chance of a child suffering from a higher hallux angle [87,96]. A higher prevalence of hallux valgus has been seen in children from China than those from Mongolia aged between seven and fourteen [98]; a hallux valgus angle of 8.2° and 6.1° was established in Chinese and Mongolian boys, respectively; a hallux valgus angle of 10° and 7.3° was discovered in Chinese and Mongolian girls. This paper compared the foot morphology of two different populations and suggested that these differences were due to the Chinese population wearing shoes with a narrow toe box [98]. Contrary to this, Hollander et al. [50] found that barefoot participants had higher hallux angles in all age groups (6–18 years old) than their shod counterparts. This finding did not support their original hypothesis. The authors concluded that barefoot children who participated in the study might have had to wear shoes as part of their school uniform, and that these shoes may have been more constrictive than those of the habitually shod individuals.

There seems to be two different issues concerning footwear and hallux valgus angle and these should not be intertwined. The first issue focuses on the effect of ill-fitting shoes and the second concerns the universal effect of shoes. Future research should aim to distinguish whether they are investigating the effects of ill-fitting shoes or shoes in general. There are limited studies that have investigated the hallux valgus angle in populations with different footwear habits, so more data is required [50,97,98]. Hollander et al. [50] is the only study that has assessed participant footwear habits, while others have simply speculated [97,98]. Ethnicity was found to significantly affect hallux valgus angle by Hollander et al. [50]. This was not investigated by other authors. Radiographic imaging remains the gold standard method for quantifying the hallux valgus angle; however, when conducting testing on children, ethical issues arise. Not only this, but radiographic imaging is expensive and not widely available. Barnicot et al. [97] explained that caution is required when comparing results collected from radiographs and footprints due to low correlations between the two methods (r = 0.56). Future research should investigate the reliability of novel techniques to quantify this structure while considering correlations with radiographic imaging. 

### 4.6. Summary and Remarks

This section of the review has revealed that footwear does influence the developing foot. The most noticeable differences were found in the development of the medial longitudinal arch, foot muscle strength and hallux valgus angle. There was some conflicting evidence regarding foot length and width, together with limitations found in some of the studies mentioned. Factors such as age, gender, ethnicity, BMI and physical activity levels were shown to influence the development of the foot and, therefore, must be controlled when trying to uncover how footwear affects foot development. Better classification of footwear habits is also necessary, as some studies have simply speculated about this information.

When it comes to the methods used to quantify the structures in the foot, there are two main problems. On one hand, the vast number of methods used to quantify the different foot structures make comparisons between different studies difficult, even though they depict a similar story. This was the case for the measurement of the medial longitudinal arch especially. Contrary to this, there is motive to investigate hallux valgus angles further; however, a more reliable and portable method should be implemented. Currently, there is not enough evidence to confidently suggest how and if footwear effects foot length and width. Moreover, the absence of studies investigating hallux valgus angles and foot strength with sufficient detail on other factors that could affect the foot structure means that more insight is needed. Future research should focus on these areas to provide clarity and clear data on populations with different footwear habits.

## 5. Conclusions

Research comparing those with different footwear habits is growing; however, the evidence is weak for its long-term effects on different foot structures. Footwear is widely regarded as an external factor that can change foot form and function. To date, most research in this field has been conducted on adults, but research based on children is growing. This narrative review aimed to synthesize research to understand what effects footwear has on the developing foot. A single study found that barefoot children between the ages of 6 and 10-years-old had wider feet than their shod counterparts. However, overall foot width was similar in both the shod and barefoot cohorts. This review found conflicting evidence regarding foot length, with two studies reporting longer feet in barefoot participants and a single study reporting the opposite. One study established that barefoot children had stronger feet during short foot exercises compared to their shod counterparts. Medial longitudinal arch height was the most popular structure investigated in the two footwear conditions and indicated that shod children had flatter feet than those who were barefoot. Greater hallux valgus angles were reported in shod populations. 

The foot is a complex structure with 26 bones, numerous ligaments, tendons and muscles and is, therefore, a challenging subject. Despite this, research on the foot continues to grow, especially in terms of the effects of footwear on its structure. This review has, however, uncovered some limitations which consequently question the strength of some of the findings reported. Firstly, some research simply speculates on the footwear habits of its participants using observations. We feel this is a major limitation due to the fact they may sometimes wear shoes—for example, when going to school. For this reason, the footwear habits of each participant need to be examined vigorously. Moreover, there is no standard method for quantifying the different structures in the foot, and the variety of research questions and specific aims make comparing results across studies a challenging task. Finally, there are many intrinsic (sex, age, genetics and BMI) and extrinsic (footwear and physical activity levels) factors that can affect growth and development. To truly understand if and how footwear habits can affect foot form and function, these factors must be controlled for. Without controlling for these factors, accurate analysis becomes impossible. Future work should continue to investigate the relationship between footwear and foot structure in children and adolescent populations and control for these potential confounding factors.

## Figures and Tables

**Table 1:** A summary of the studies examined relating to the development of foot length and width.

Author	Study Type	Participants & Age Range	Mean Age (±SD)	Measures	Main Findings
Volpon et al. [29]	Cross-sectional	338 boys 334 girls 0–15 years	N/A	Plantar surface length	From infancy until 12 years old, there was no significant difference in foot length growth in boys and girls. After the age of 12, girls’ growth plateaued, whilst the boys continued to grow. The highest increase in foot length was until the age of 3 (mean ≈ 5.3 cm of growth).
Bosch et al. [19]	Longitudinal	16 boys 20 girls 0–10 years	14.6 ± 1.8 months 122.8 ± 2 months	Foot length	A continuous increase in foot length between the ages of 1 and 10 years. Foot length did not differ significantly between boys and girls between 1 and 10 years old.
Gould et al. [25]	Longitudinal	107 boys and girls 1–5 years	N/A	Foot length Foot width	Children’s feet grow in growth spurts, and these can take place at different ages. 54% of toddlers aged 1 and younger required a shoe change every 2 months or less. 40% of 1- to 2-year-olds needed a shoe change every 2–3 months. The growth rate slows down from the ages of 2 to 5, and the children require a shoe change every 4 months or longer.
Mickle et al. [21]	Cross-sectional	36 boys 52 girls 3–5 years	4.2 ± 0.6 years	Foot length Foot width	Foot length (B: 16.2 ± 1 cm; G: 15.6 ± 1.2 cm). Foot width (B: 6.6 ± 0.3 cm; G: 6.5 ± 0.4 cm). No gender differences in normalised foot length (*p* = 0.015).
Cheng et al. [32]	Cross-sectional	1408 boys 1421 girls 3–18 years	N/A	Foot length Foot width	Foot length increased in boys between age 3 and 18 (3 years: 15.6 ± 0.8 cm; 18 years: 25.8 ± 1.5 cm). Foot length increased in girls between age 3 and 17 (3 years: 15.3 ± 0.9 cm; 17 years: 23.4 ± 1.3 cm). Foot width increased in boys aged between 3 and 17 (B 3 years: 5.8 ± 0.3 cm; B 17 years: 9.4 ± 0.3 cm and girls aged 3 to 13; G 3 years 5.9 ± 0.3 cm; G 13 years: 8.3 ± 0.5 cm). A more rapid growth rate in length was witnessed in boys between the ages of 3 to 15 compared to girls (plateaued at 25.3 cm and 2.5 cm longer than girls).
Bari et al. [27]	Cross-sectional	129 boys 174 girls 5–6 years	N/A	Plantar surface length and width	Boys had longer feet than girls (B: 17.8 ± 1 cm; G: 17.8 ± 1. cm). Boys had wider feet than girls (B: 7.2 ± 0.5 cm; G: 7.1 ± 0.5 cm). Children aged 6 years had larger foot measurements than 5-year-olds.
Chen et al. [26]	Cross-sectional	549 boys 475 girls 5–13 years	N/A	Foot length Foot width	Foot length (B: 21 ± 2.1 cm; G: 20.7 ± 2.2 cm). Foot width (B: 8.8 ± 0.8 cm; G: 7.9 ± 0.8 cm).
Müller et al. [28]	Cross-sectional	3738 boys 4050 girls 1–13 years	7.2 ± 2.9 years	Foot length Foot width	Foot length increased with age (1 year: 13.1 ± 1.6 cm; 13 years: 24.4 ± 3 cm). Foot width increased with age (infants: 5.7 ± 0.8 cm; 13 years: 8.9 ± 1.3 cm).
Morrison et al. [18]	Cross-sectional	90 boys 110 girls 9–12 years	N/A	Foot length Foot width	From the ages of 10–12 years old, boys usually had longer and wider feet than girls. An increase in foot length and width was present between the ages of 9 and 12 years. Foot length (age 12; B: 23.4 ± 1.2 cm; G: 23.2 ± 1.3 cm). Foot width (age 9; B: 7.9 ± 0.5 cm; G: 8.1 ± 0.5 cm).
Xu et al. [31]	Cross-sectional	1252 boys 1274 girls 13–18 years	N/A	Foot length Foot width	Length measurements increased significantly between 13 and 14 years in boys (13 years: 24.6 ± 1.3 cm; 14 years: 25 ± 1.3 cm) and 14–15 years in girls (14 years: 23.2 ± 0.9 cm; 15 years: 23.7 ± 1 cm). Length and width measurements plateaued by the age of 15 in girls and 16 in boys.
Waseda et al. [20]	Cross-sectional	5311 boys 4844 girls 6–18 years	N/A	Foot length	Foot length in boys increased significantly with age and almost plateaued at 14 years (6 years: 18.4 ± 1 cm; 14 years: 25.1 ± 1.1 cm; *p* < 0.01). Foot length in girls increased significantly with age and almost plateaued at 13 years (6 years: 18.1 ± 0.9 cm; 13 years: 23.1 ± 1 cm).
Delgado-Abellán et al. [23]	Cross-sectional	497 boys 534 girls 6–12 years	N/A	Foot length Foot width	The biggest difference between girls and boys foot length was present between the ages of 8–10 years. (B 8 years: 21.2 ± 1.1 cm; G 8 years: 20.6 ± 1.1 cm; B 10 years: 23 ± 1.2 cm; G 10 years: 22.6 ± 1.4 cm). No gender differences were seen in normalised foot length. Gender differences in normalised foot width.
Xu et al. [30]	Cross-sectional	1240 boys 1303 girls 7–12 years	N/A	Foot length Foot width	Boys had significantly longer feet than girls aged 12 (B: 24.1 ± 13.2 cm; G: 23 ± 9.4 cm; *p* < 0.05; ES > 0.8). Foot length and body height were linearly associated with each other. The most prominent growth rate in foot length for girls occurred at 7–8 (5.3%) and 8–9 years (5.2%). The most notable growth rate in foot length for boys occurred at 8–9 (5.6%) and 10–11 years (6%).
Blais et al. [22]	Longitudinal	285 boys 227 girls 1–18 years	N/A	Foot length	The highest growth rate was seen through infancy to 5 years old (B 1 years: 10.9 cm; G 1 years: 10.5 cm; B 5 years: 19.2 cm; G 5 years: 18.9 cm). An annual increase of 0.9 cm was seen from 5 years until 12 years in girls and 5 years until 14 years in boys. At the age of 12, the average length of the foot was similar in boys and girls (23.5 cm and 23.2 cm, respectively). After the age of 12, boys feet continued to grow until the age of 16 (an increase of 2.7 cm). After 12 years old, girls’ feet slowly continued to grow until 14 years (a rise of 0.8 cm).
Anderson et al. [24]	Longitudinal	285 boys 227 girls 1–18 years	N/A	Foot length	The growth rate was fastest until the age of 5 years old (B 1 years: 11.9 cm; B 5 years: 17.3 cm; G 1 years: 11.9 cm; G 5 years: 17.1 cm). Annual growth of 0.9 cm was seen between the ages of 1 and 5 years. Under the age of 13, the mean foot length in girls and boys was similar; however, girls usually had slightly shorter feet. Girls’ feet grew very little after age 12 (0.7 cm on average); however, boys feet grew 2.6 cm on average after age 12. 75% of girls’ feet had reached mature length by age 14. 70% of boys’ feet had reached mature length by age 16. Boys’ feet were about 2.5 cm longer than girls at the end of growth.

Abbreviations: B—Boys; G—Girls; N/A—Not Available.

**Table 2:** A summary of the measures used to quantify the medial longitudinal arch.

Measurement	Equipment	Position	Diagram	Calculation
Arch Index (AI)	Foot scan Inked footprint	Bilateral weight-bearing	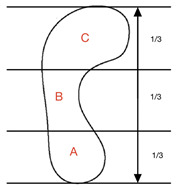	AI = B/A + B + C
Clarke’s/Footprint Angle (CFA)	Foot scan Inked footprint	Bilateral weight-bearing	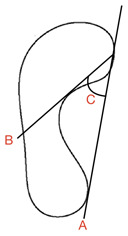	CFA = angle C
Chippaux-Smirak Index (CSI)	Foot scan Inked footprint	Bilateral weight-bearing	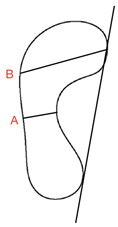	CSI = (A/B × 100%)
Staheli Index (SI)	Foot scan Inked footprint	Bilateral weight-bearing	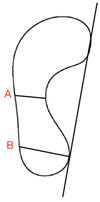	SI = (A/B × 100%)
Arch Height Ratio (AHR)	Callipers	Bilateral weight-bearing	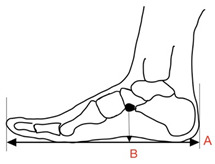	AHR (%) = B × 100/A

**Table 4:** A summary of the studies examined relating to footwear habits and foot length and width.

Author	Study Type	Ethnicity	Participants & Age Range	Mean Age (±SD)	Method of Analysis	Main Findings
Hollander et al. [50]	Cross-sectional	Included ethnicity as a confounding variable, however, did not specify what the ethnicities were.	810 male and female 6–18 years	11.99 ± 3.33 years	Foot width Foot length	Foot length and foot width increased with age. Longer feet found in the age groups 6–10 and 14–18 for habitually barefoot participants (HB 6–10 years: 20.90 ± 1.38 cm; HS 6–10 years: 20.62 ± 1.45 cm; HB 14–18 years: 25.76 ± 1.94 cm; HS 14–18 years: 24.94 ± 1.65 cm). Wider feet were found in the age group 6–10 for habitually barefoot participants (HB 6–10 years: 8.23 ± 0.49 cm; HS 6–10 years: 8.08 ± 0.51 cm).
Kusumoto et al. [76]	Cross-sectional	Filipino Japanese	582 male 541 female 7–18 years	N/A	Foot length Foot width	Tokyo boys had longer feet than Isabela boys aged 8–12 years (T 8 years: 19.6 ± 1.2 cm; I 8 years: 18.4 ± 0.9 cm; T 12 years: 22.8 ± 1.1 cm; I 12 years: 21.5 ± 1.7 cm). Tokyo girls had longer feet than Isabela girls aged 7–11 years (T 7 years: 18.6 ± 1.1 cm; I 7 years: 17.6 ± 0.6 cm; T 11 years: 21.9 ± 1.3 cm; I 11 years: 20.7 ± 1.3 cm) The relatively wide feet present in Isabela children was hypothesised to be due to a difference in their foot shape.
Wolf et al. [75]	Cross-sectional	N/A	18 male and female 6–10 years	8.2 ± 0.7 years	Foot width	A study investigating the acute changes in foot width in different footwear. Foot width showed a variation of 10% during the gait cycle when barefoot. Foot width variation was reduced when wearing shoes to 4% in shoe 1 and 6% in shoe 2.
Mauch et al. [49]	Cross-sectional	N/A	448 male 562 female 3–12 years	Australian 4.3 ± 0.6 & 9.6 ± 1.4 years German 4.2 ± 0.7 & 9.6 ± 1.4 years	Foot length	The German preschool children’s feet were significantly longer than their Australian counterparts (G: 16.8 ± 1.1 cm; A: 15.8 ± 1.1 cm) When matched for height, differences in foot length were still seen. There was no difference in foot length in the primary school children (G: 22.1 ± 1.5 cm; A: 21.9 ± 1.6 cm). The foot width was within the measurement error and was not discussed.

Abbreviations: HB—Habitually Shod; HB—Habitually Barefoot; T—Tokyo; I—Isabella; G—German; A—Australian; N/A—Not Available.

**Table 5:** A summary of the studies examined relating to footwear habits and medial longitudinal arch development.

Author	Study Type	Ethnicity	Participants & Age Range	Mean Age (±SD)	Method of Analysis	Main Findings
Mauch et al. [49]	Cross-sectional	N/A	448 male 562 female 3–12 years	Australian- 4.3 ± 0.6 & 9.6 ± 1.4 years German- 4.2 ± 0.7 & 9.6 ± 1.4 years	Footprint angle Chippaux–Smirak Index	There was a significant difference in arch height in preschool-aged children. The Australian preschool children displayed higher arches than those from Germany (39.4° compared to 24°). No significant differences in arch height in primary school-aged children.
Hollander et al. [50]	Cross-sectional	Included ethnicity as a confounding variable, however, did not specify what the ethnicities were.	810 male and female 6–180 years	11.99 ± 3.33 years	Static arch height index Dynamic arch index	An increased static arch height was seen in children and adolescents who grow up barefoot. The findings suggested that habitual footwear use influences the development of the medial longitudinal arch. The arch index only differed in the 10–14 age group (flatter arch in the shod participants).
Rao et al. [42]	Cross-sectional	Rural Indian	1237 male 1063 female 4–13 years	NR	Footprint angle	At age ten, the flat foot prevalence was very low in the barefoot participants and high in the shod participants. Closed-toe shoes seemed to inhibit the development of the arch more than slippers or sandals.
Aibast et al. [86]	Cross-sectional	Kenyan	39 male 39 female 12–18 years	Study 1; 15.1 ± 1.4 years Study 2; 15.5 ± 1.2 & 15.4 ± 1.2 years	Arch height ratio	The habitually barefoot participants had higher medial longitudinal arches. Using the criteria for arch height ratio, habitually barefoot participants would be classified as having high arches.
Sachithanandam et al. [88]	Cross-sectional	South Indian	1846 male and female 16–65 years	NR	Footprint angle	The findings of this study suggested that there is an association between wearing shoes in early life and flat foot.
Echarri et al. [57]	Cross-sectional	Congolese	945 male 906 female 3–12 years	NR	Clarke’s angle Chippaux–Smirak Index Staheli Arch Index	There was a greater proportion of flat feet in the urban environment. This study showed that the development of the medial longitudinal arch is influenced by age, sex and footwear.
Tong et al. [85]	Longitudinal	Not clear	52 male 59 female 7–9 years	6.9 ± 0.3 years 8.7 ± 0.3 years	Dynamic arch index	Participants who wore closed-toe shoes displayed the flattest arch.

Abbreviations: NR—Not Recorded.
